# Supplementary material for: Purification, Characterization and Evaluation of Inhibitory Mechanism of ACE Inhibitory Peptides from Pearl Oyster (Pinctada fucata martensii) Meat Protein Hydrolysate
Source: Mar Drugs. 2019 Aug 8;17(8):463. doi: 10.3390/md17080463 (PMC6723713; doi:10.3390/md17080463)
Supplement: Supplementary file 1 [file marinedrugs-17-00463-s001.pdf]

Supplementary Materials:

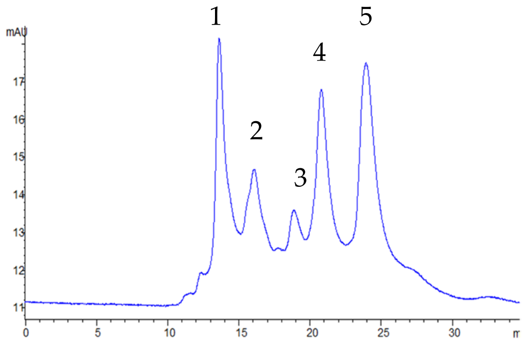

(**a**)

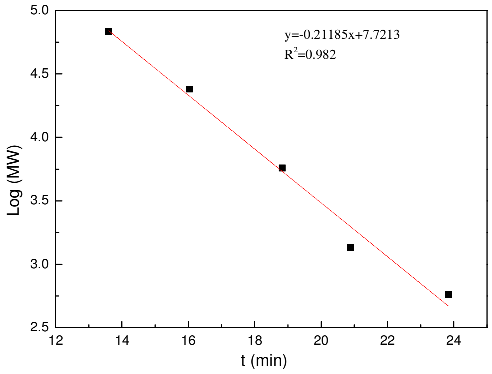

(**b**)

Figure S1. GPC chromatograms of standard materials (**a**) and standard curve (**b**) obtained from standard materials: (1) BSA (66 kDa), (2) trypsin inhibitor (24 kDa), (3) human insulin (5808 Da), (4) vitamin B_{12} (1355 Da), and (5) FFVAP (577.92 Da).
